# *Bacillus megaterium* NCT-2 agent alters soil nutrients, vegetable quality, and root microecology in secondary salinized soil

**DOI:** 10.3389/fmicb.2025.1543933

**Published:** 2025-04-22

**Authors:** Yimin You, Shitong Li, Liran Wang, Xiyang Zhao, Dan Zhang, Shaohua Chu, Xijia Yang, Pei Zhou

**Affiliations:** ^1^Jilin Provincial Key Laboratory of Tree and Grass Genetics and Breeding, College of Forestry and Grassland Science, Jilin Agricultural University, Changchun, China; ^2^Key Laboratory of Urban Agriculture, Ministry of Agriculture and Rural Affairs, Shanghai, China; ^3^School of Agriculture and Biology, Shanghai Jiao Tong University, Shanghai, China; ^4^Bor S. Luh Food Safety Research Center, Shanghai Jiao Tong University, Shanghai, China; ^5^Shanghai Yangtze River Delta Eco-Environmental Change and Management Observation and Research Station, Ministry of Science and Technology, Ministry of Education, Shanghai, China

**Keywords:** secondary salinized soil, microbial agent, soil nutrient, vegetable quality, microbial community

## Abstract

Microbial remediation technology has the characteristics of high efficiency and environmental protection, which has attracted attention. However, there is complexity in the microorganism-soil–plant system. The effects of microbial agents on soil nutrients, plant quality, rhizosphere, and endophytic microorganisms are still unclear. Here, we demonstrate the application of *Bacillus megaterium* NCT-2 as a multifunctional agent that concurrently addresses salinization-driven nutrient imbalances and reshapes keystone microbial taxa to restore soil–plant homeostasis. The results showed that NCT-2 agent improved the soil nutrients, reduced the loss of nitrogen and sulfur, increased the content of available phosphorus, and decreased the electrical conductivity. The agent increased the number of bacteria and fungi in the soil. Meanwhile, NCT-2 agent improved the vegetable quality and yield. Specifically, the NCT-2 agent significantly increased the aboveground fresh weight, underground fresh weight, total flavonoids, antioxidant enzyme activity, ascorbic acid, Cu, Zn, Fe, P, and K in lettuce, while significantly reduced nitrate. The chlorophyll a, chlorophyll b, carotenoids, and total chlorophyll were significantly increased by the agent. Critically, high-throughput sequencing revealed NCT-2-driven enrichment of stress-resilient taxa (e.g., *Firmicutes*, *Acidobacteria*) and functional synergists (e.g., *Acetobacter*), which correlated with soil nutrient fluxes and plant antioxidant capacity. By decoupling the interplay between microbial community restructuring and systemic remediation outcomes, this work establishes a novel framework for leveraging keystone taxa to optimize salinized agroecosystems.

## 1 Introduction

Greenhouse cultivation has rapidly developed into one of the main industries in agriculture. Its production mode is mainly high input and high output, which leads to the increasingly serious soil secondary salinization (Zhang et al., 2021). Secondary salinized soil has high salt concentration and high osmotic pressure between pore water and roots, which inhibits water transport from soil to plants. There are adverse effects on soil physicochemical properties, microbial processes and plant growth (Baek and Yang, 2009; Cho et al., 2009). Microbial remediation can remove pollutants and salt ions with low input, no pollution and high efficiency (Ravindran et al., 2007). The method also has the potential to restore soil quality and function. Therefore, microbial remediation is receiving global attention (Wang et al., 2022). At present, most studies only focused on the removal efficiency of specific pollutants and the microbial community structure of single niche during microbial remediation. Few studies have investigated the combined effects of microbial application on soil nutrients, plant quality, and rhizosphere and root microbial communities.

*Bacillus megaterium* is an important rhizosphere bacterium with a wide ecological range, which has become a research hotspot of restoration stress (Wu et al., 2017; Adrees et al., 2019). For example, *Bacillus megaterium* was used as a tool in bioremediation technology to degrade pyrene and other polycyclic aromatic hydrocarbons (Meena et al., 2016). *Bacillus megaterium* can be used as a salt inhibitor to improve soil salinity, nutrients and plant biomass (Abdel Motaleb et al., 2020). In addition, this strain can produce proline and indoleacetic acid (Marulanda et al., 2009). Therefore, it is certain that a specific strain of *Bacillus megaterium* is able to improve some kind of contaminated soil environment.

Rhizosphere and root environment are the key hotspots of biogeochemical transformation and microbial interactions. In the narrow area of the rhizosphere, a large number of microorganisms significantly affect plant growth, nutrition and health (Hou et al., 2021). Some rhizosphere microorganisms can enter the roots to form endophytic microbiomes, which affect plant community structure, growth and resistance (Feng et al., 2022). In microbial remediation, the combined effects of root exudations and salt stress reduction cause variation in soil physicochemical parameters, which may lead to change in microbial interactions at rhizosphere and root (Wang et al., 2022). Microorganisms adapted to specific environments often have similar resource utilization strategies. Many microorganisms with similar ecological functions may co-exist (Luo et al., 2022). Thus, the addition of specific functional microorganisms may alter environmental parameters and microbial interactions, thereby promoting specific functions of microorganisms.

*Bacillus megaterium* NCT-2 was screened in our laboratory. The strain can efficiently transform nitrate and reduce nitrite accumulation to remediate secondary salinized soil (You et al., 2021). While nitrate metabolism represents its primary remediation pathway, recent studies on phylogenetically related *Bacillus strains* suggest that microbial salt stress mitigation often involves synergistic effects across multiple functional axes. For instance: *Bacillus velezensis JB0319* improves K^+^/Na^+^ homeostasis by upregulating HKT1 transporters, reducing Na^+^ accumulation in lettuce under salinity (Bai et al., 2023); *Bacillus aryabhattai SRB02* enhances root biomass via glycine betaine synthesis, mitigating osmotic stress in salt-affected plant (Park et al., 2017); *Bacillus licheniformis HSW-16* alleviates salt-induced ethylene stress through ACC deaminase activity, boosting photosynthetic efficiency in plant (Singh and Jha, 2016). Furthermore, NCT-2 inoculation may indirectly modulate lettuce salt tolerance by altering rhizosphere microbial community composition and functional potential, as observed in *Bacillus* spp.-mediated enrichment of salt-tolerant taxa and suppression of pathogenic fungi (Zhang et al., 2023). Although the specific contributions of NCT-2 beyond nitrate transformation require further mechanistic validation, these findings collectively highlight the importance of evaluating remediation outcomes at systemic levels. Our study therefore integrates soil nutrient analysis, lettuce quality assessment, and rhizosphere microbiome profiling to comprehensively quantify NCT-2’s restoration efficacy in secondary salinized ecosystems.

Lettuce, a globally cultivated leafy vegetable with high economic value in greenhouse production, was selected as the model plant for three key reasons: (1) Its shallow root system and rapid growth cycle make it highly sensitive to soil salinity changes; (2) As a nitrate-accumulating species, lettuce quality is directly impacted by nitrogen metabolism imbalances in salinized soils (Broadley et al., 2003); (3) Previous studies have established its utility as a bioindicator for microbial remediation efficiency in protected cultivation systems (Bai et al., 2023).

In order to analyze the application prospects of NCT-2 agent, elemental analysis, ionomics, and amplicon sequencing were used to solve three problems. (i) Soil nutrients and lettuce quality were altered by microbial agents. (ii) Rhizosphere and root microorganisms are affected by microbial agents. (iii) The correlation between soil nutrients, plant quality, and core microbial communities is revealed.

## 2 Materials and methods

### 2.1 Soil sampling

The secondary salinized soil was selected by random sampling method, and the soil depth was 0–20 cm (Cui et al., 2023; Yang et al., 2023). Random stratified sampling design to ensure representativeness in heterogeneous fields. 0–20 cm sampling depth as the standard for topsoil analysis in croplands, aligning with the user’s methodology (Cui et al., 2023; Yang et al., 2023). The sampling place was Vegetable Production and Marketing Cooperative, Minhang District, Shanghai, China (121°33 “14” E, 31°0 “3” N). Basic soil properties are determined and shown in Supplementary Table 1. Specific sample testing methods were shown in Supplementary materials and methods 2.1.

### 2.2 Preparation of microbial agent

In our laboratory, *Bacillus megaterium* NCT-2 was obtained from secondary salinization soil. To analyze the strain’s ability to metabolize nitrate, the strain was cultured in a medium where mineral nitrogen was supplied as KNO_3_. After 24 h of cultivation at 35°C and 180 rpm, NCT-2 seed solution was obtained. The strain was cultured using the same culture medium as the fermentation medium and humic acid was used as the carrier to prepare the microbial agent (You et al., 2021; You et al., 2022a; You et al., 2022b).

The mineral salt medium had a composition in g L^−1^ of: KNO_3_, 1 g; KCl, 1 g; FeSO_4_·7H_2_O, 0.01 g; MgSO_4_·7H_2_O, 0.5 g; CaCl_2_, 0.01 g; KH_2_PO_4_, 0.5 g; glucose, 10 g. The pH of the medium was adjusted to 7.

### 2.3 Soil and pot experiments

Air-dried soil samples were homogenized and oven-dried at 105°C for 48 h to determine baseline moisture content. For pot setup, 1.8 kg (dry weight equivalent) of fresh soil was weighed (±0.01 g) into 2 kg capacity pots. Soil moisture was uniformly adjusted to 60% water-holding capacity (WHC) using deionized water. Microbial inoculants were applied at a concentration of ≥2 × 10^8^ CFU g^−1^ (verified by plate counting) and combined with humic acid as specified in Table 1, including a humic acid-only control to isolate NCT-2-specific effects. Ten biological replicates were maintained per treatment.

**Table 1:** The name of treatment groups and samples.

Treatments	The name of the soil samples	The name of the vegetable samples
Secondary salinized soil	CK	CK1
Secondary salinized soil with only humic acid (equal to the amount contained in the NCT-2 agent)	HA	HA2
Secondary salinized soil with NCT-2 agent	NCT-2	NCT3

The experiment was conducted in a controlled greenhouse (21–30°C, 70–80% relative humidity, 16 h light/8 h dark photoperiod) over a 30-day remediation phase. Soil moisture was replenished every 48 h via deionized water supplementation to compensate for evaporation. Lettuce (*Lactuca sativa* cv. large quick-growing) seedlings were transplanted into prepared pots after 10 days of pre-germination and cultivated for an additional 30 days under standardized conditions. This design ensured systematic evaluation of NCT-2-mediated remediation while accounting for soil–plant-microbe interactions.

### 2.4 Soil nutrient measurement

Soil samples were collected at 0 days (immediately after amendment application) and 30 days post-treatment. The soil was collected for nutrient determination. The contents of total nitrogen (TN), total carbon (TC), organic carbon (TOC) and total sulfur (TS) were determined by elemental analyzer (ThermoFisher, Germany). Determination of available phosphorus (P) by plasma emission spectrometer. Soil electrical conductivity (EC) was determined using conductivity meter (ThermoFisher, Germany). Bacterial and fungal copy numbers were determined by real-time fluorescent quantitative PCR. Primers were shown in Supplementary Table 2. Detailed methods were presented in Supplementary materials and methods 2.1 and 2.2.

### 2.5 Determination of vegetable quality

After 30 days, lettuce plants were thoroughly washed with deionized water, blotted dry with filter paper, and separated into underground (roots) and overground (shoots) parts for fresh weight measurement. The following quality parameters were analyzed:

Ascorbic acid: Fresh lettuce tissue (5 g) was homogenized in 20 mL 2% (w/v) oxalic acid solution. The homogenate was centrifuged at 8,000 × g for 15 min. The supernatant was mixed with 0.05% (w/v) 2,2′-bipyridyl and 0.1% (w/v) FeCl₃, followed by addition of 1 mL xylene. Absorbance was measured at 500 nm using a microplate reader (Azam et al., 2013).

Total phenols: Extracted with 1% HCl-methanol (1:4, v/v) for 24 h in darkness. The supernatant was reacted with Folin–Ciocalteu reagent (1:10, v/v) and 7.5% Na₂CO₃ (1:2, v/v). After 30 min incubation, absorbance was read at 760 nm, with gallic acid as the standard (Sarker and Oba, 2018).

Flavonoids: The extract (500 μL) was mixed with 1.5 mL methanol, 0.1 mL 10% AlCl₃, 0.1 mL 1 M CH₃COOK, and 2.8 mL H₂O. After 30 min at 25°C, absorbance was measured at 415 nm, and quantified using quercetin calibration (Sarker and Oba, 2018).

Total soluble sugars: Quantified by phenol-sulfuric acid method: 100 μL extract was mixed with 5% phenol (100 μL) and concentrated H₂SO₄ (1 mL), incubated for 10 min, and read at 485 nm (Wang et al., 2021).

Fructose, glucose, sucrose: Ultrasonically extracted with ultrapure water (100 mL, 30 min), centrifuged at 10,000 × g for 5 min, and analyzed via HPLC (Shimadzu LC-20 AD) with a refractive index detector and Hi-Plex H column (Wang et al., 2021).

Protein content and antioxidant enzymes: Determined using commercial ELISA kits (Sigma-Aldrich BC0175 for SOD, BC0200 for CAT) following manufacturer protocols.

Nitrate content: Homogenate (10 g) was boiled in 50 mL deionized water for 15 min, filtered, and analyzed spectrophotometrically at 540 nm after cadmium reduction (Yu et al., 2018).

Photosynthetic pigments: Extracted with ethanol:acetone (1:1, v/v) for 12 h in darkness. Absorbance was measured at 663 nm (chlorophyll a), 645 nm (chlorophyll b), and 470 nm (carotenoids) (Nawaz et al., 2018; Sharma et al., 2020).

Mineral elements: Microwave-digested with HNO₃:HClO₄ (4:1, v/v) and H₂O₂, then analyzed by ICP-OES (iCAP 7400, ThermoFisher) (Khaliq et al., 2019; Mizushima et al., 2019).

Full protocols are provided in Supplementary materials and methods 2.3.

### 2.6 High-throughput sequencing

#### 2.6.1 DNA extraction and PCR amplification

The names of high-throughput sequenced samples from rhizosphere soil and roots were shown in Table 2. The genomic DNA was extracted from enrichment samples using FastDNA^®^ Spin Kit for soil (MP Biomedicals, GA, United States) according to manufacturer’s instructions. The DNA extract was checked on 1% agarose gel, and DNA concentration and purity were determined with NanoDrop 2000 UV–vis spectrophotometer (Thermo Scientific, Wilmington, United States). The hypervariable region V3–V4 of the bacterial 16S rRNA gene were amplified with primer pairs 338F (5′-ACTCCTACGGGAGGCAGCAG-3′) and 806R (5′-GGACTACHVGGGTWTCTAAT-3′) by an ABI GeneAmp^®^ 9700 PCR thermocycler (ABI, CA, United States). The PCR amplification of 16S rRNA gene was performed as Supplementary materials and methods 2.4. The PCR product was extracted from 2% agarose gel and purified using the AxyPrep DNA Gel Extraction Kit (Axygen Biosciences, Union City, CA, United States) according to the manufacturer’s instructions and quantified using Quantus™ Fluorometer (Promega, United States) (You et al., 2021; You et al., 2022a; You et al., 2022b).

**Table 2:** Sample names of microbial diversity in rhizosphere soil and root.

Treatments	Sample name of rhizosphere soil	Sample name in root
Secondary salinized soil	CKa	CK
Secondary salinized soil with only humic acid (equal to the amount contained in the NCT-2 agent)	HUa	HU
Secondary salinized soil with NCT-2 agent	NCTa	NCT

#### 2.6.2 Illumina MiSeq sequencing

Purified amplicons were pooled in equimolar and paired-end sequenced on an Illumina MiSeq PE300 platform/NovaSeq PE250 platform (Illumina, San Diego, United States) according to the standard protocols by Majorbio Bio-Pharm Technology Co. Ltd. (Shanghai, China) (You et al., 2021; You et al., 2022a; You et al., 2022b). The raw reads were deposited into the NCBI Sequence Read Archive (SRA) database (Accession Number: PRJNA1081162). The detailed processing of sequencing data was shown in the Supplementary materials and methods 2.5.

All data analysis methods are presented in Supplementary materials and methods 2.6.

### 2.7 Data analysis

A two-way analysis of variance (ANOVA) was conducted with IBM SPSS Statistics version 26.0 (Chicago, United States) to examine the effects of two independent variables, namely age (1, 5, and 11) and microbial environment (non-rhizosphere, rhizosphere, and roots) on nutrient cycle. The microbial taxonomic composition and chao index was analyzed using an R package (Vegan) version 3.2.0. All results were reported as the mean ± standard deviation.

## 3 Results

### 3.1 Impact of NCT-2 agent on microbial diversity

**Figure 1:**
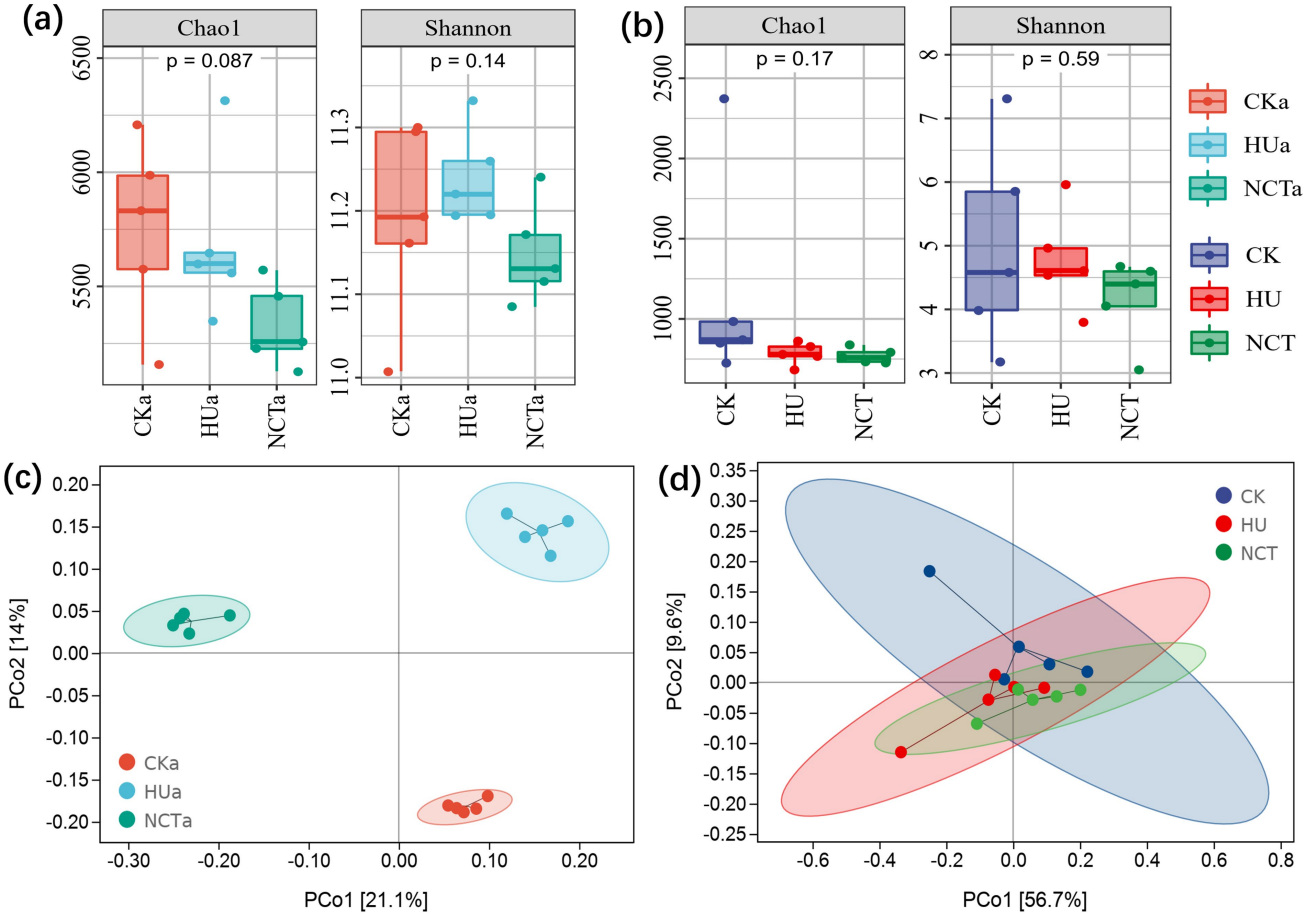
Effect of NCT-2 agent on α and β diversity of microbial community. **(a)** Chao index and Shannon index of soil microbial community in rhizosphere; **(b)** Chao index and Shannon index of microbial community in root; **(c)** Beta diversity of microbial communities in rhizosphere; **(d)** Beta diversity of microbial communities in root.

High-throughput sequencing was used to characterize the effects of NCT-2 agent on α and β diversity in rhizosphere and root microbial communities. The effects of humic acid and NCT-2 agent on microbial richness and diversity were not significant in the rhizosphere soil and roots (Figures 1a,b). Analysis of β diversity showed that humic acid and NCT-2 agent significantly affected the microbial community structure of rhizosphere (Figure 1c). PCo1 and PCo2 indicated that NCT-2 agent had a greater impact on microbial communities (Figure 1c). In addition, both treatments had little effect on the composition of the microbial community in the roots (Figure 1d).

### 3.2 Microbial agents changed the composition of microbial communities in rhizosphere and root

**Figure 2:**
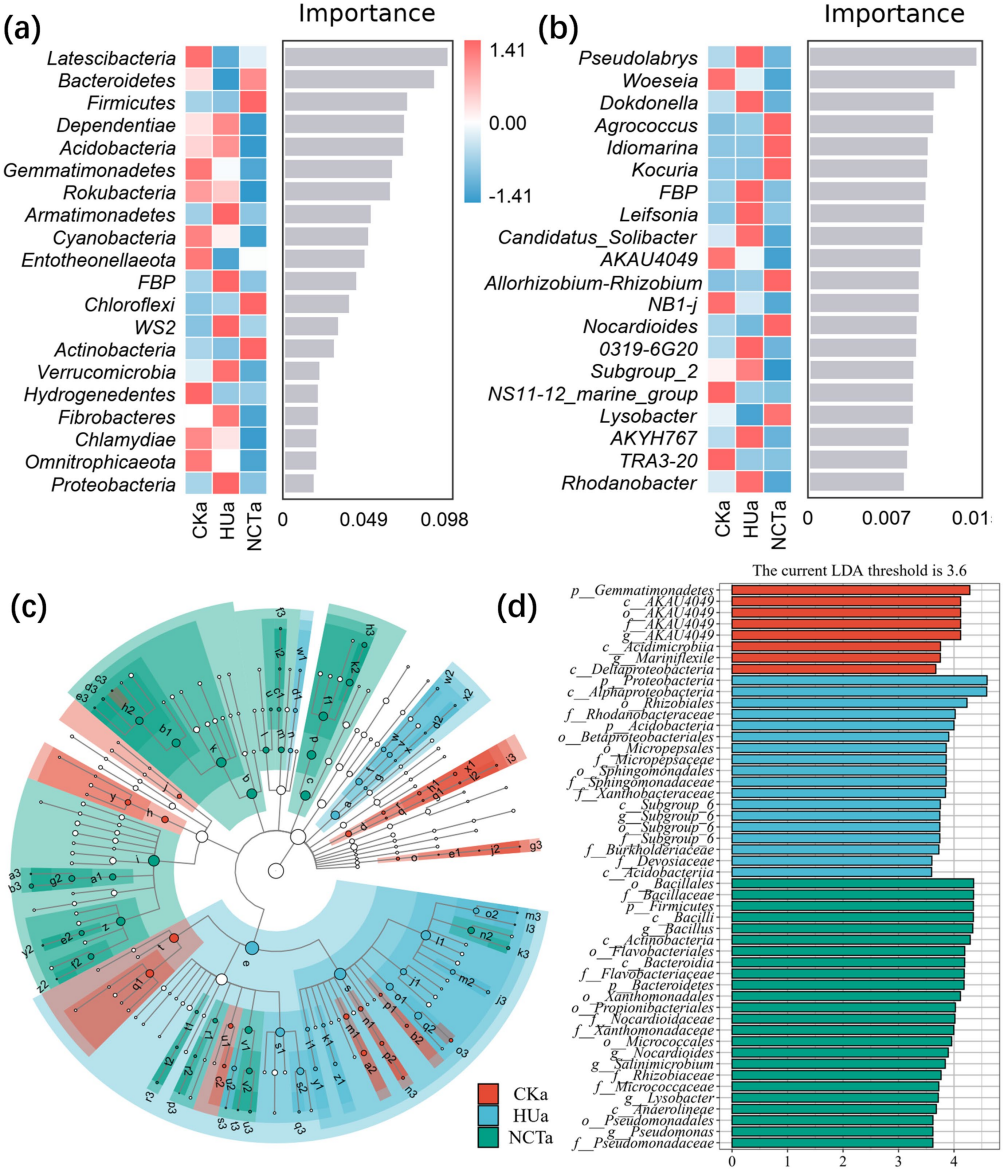
Response of microbial community composition to NCT-2 agent in rhizosphere. **(a)** Random forest analysis at the phyla level; **(b)** Random forest analysis at the genus level; **(c)** Branch graphs based on LEfSe analysis; **(d)** Column chart based on LEfSe analysis.

In depth analysis revealed that NCT-2 agent altered the composition of rhizosphere and endophytic microbial communities. The *Proteobacteria*, *Actinobacteria*, *Bacteroidetes*, *Gemmatimonadetes*, *Firmicutes*, *Acidobacteria*, and *Chloroflexi* of rhizosphere microbial communities were the main phyla, accounting for approximately 90% (Supplementary Figure S1). The impact of microbial agents on microbial communities at the phyla and genus levels was analyzed through random forest analysis. In the rhizosphere soil, phyla level analysis revealed the 20 most affected bacteria, including *Latescibacteria*, *Bacteroidetes*, *Firmicutes*, *Dependentiae*, and *Acidobacteria* (Figure 2a). *Pseudolabrys*, *Woeseia*, *Dokdonella*, *Agrococcus*, and *Idomanna* genera were marker species for inter treatment differences (Figure 2b). Combined with LEfSe analysis, 49 significantly different species were identified (Figures 2c,d). In the rhizosphere microbiome of control group, *p__Gemmatimonadetes*, *c__AKAU4049*, *c__Acidimicrobiia*, *g__Mariniflexile* and *c__Deltaproteobacteria* were the most significant biomarker taxa. In humic acid treatment, *p__Proteobacteria*, *c__Alphaproteobacteria*, *o__Rhizobiales*, *f__Rhodanobacteraceae* and *p__Acidobacteria* were the most significant biomarker taxa. In the treatment of NCT-2 agent, *p__Firmicutes*, *g__Bacillus*, *c__Actinobacteria*, *g__Nocardioides* and *g__Pseudomonas* were the most significant biomarker taxa (Figures 2c,d).

**Figure 3:**
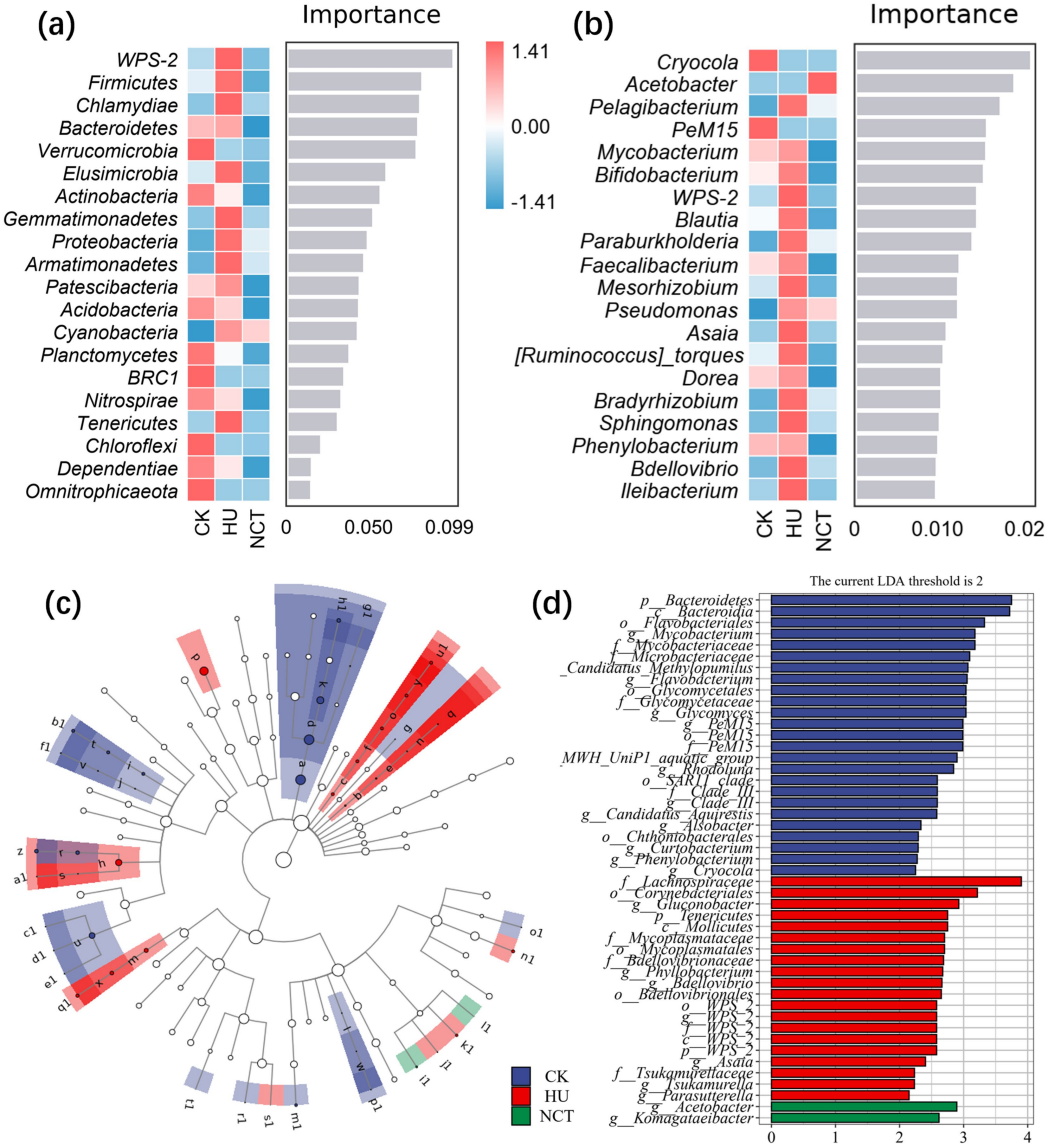
Response of microbial community composition to NCT-2 agent in root. **(a)** Random forest analysis at the phyla level; **(b)** Analysis of random forest at the genus level; **(c)** Branch graphs based on LEfSe analysis; **(d)** Column chart based on LEfSe analysis.

The *Proteobacteria*, *Cyanobacteria*, and *Actinobacteria* in the root were the main phyla, accounting for more than 90% (Supplementary Figure S1). In the root, *WPS-2*, *Firmicutes*, *Chlamydiae*, *Bacteroidetes*, and *Verrucomicrobia* were the most affected phyla (Figure 3a). *Cnyocola*, *Acetobacter*, *Pelagibacterium*, *PeM15*, and *Mycobacterium* were the most affected genera (Figure 3b). Furthermore, 47 significantly different species were identified through LEfSe analysis (Figures 3c,d). In the control group, the most significant biomarker taxonomic units were identified as *p_ Bacteroidetes*, *c_ Bacteroidia*, *o_Flavobacteria*, *g_ Mycobacterium*, *f_ Mycobacteriaceae*. In the treatment of humic acid, the most significant taxonomic units for biomarkers were *f_ Lachnospiraceae*, *o_ Corynebacteria* and *g_ Gluconobacter*. In NCT-2 agent treatment, *g_ Acetobacter* and *g_ Komagataeibacter* were the most significant biomarker taxonomic units (Figures 3c,d). These results indicated that NCT-2 agent changed the composition of rhizosphere and root microbial community, and had a greater effect on rhizosphere microorganisms.

### 3.3 Effect of NCT-2 agent on soil nutrients

**Figure 4:**
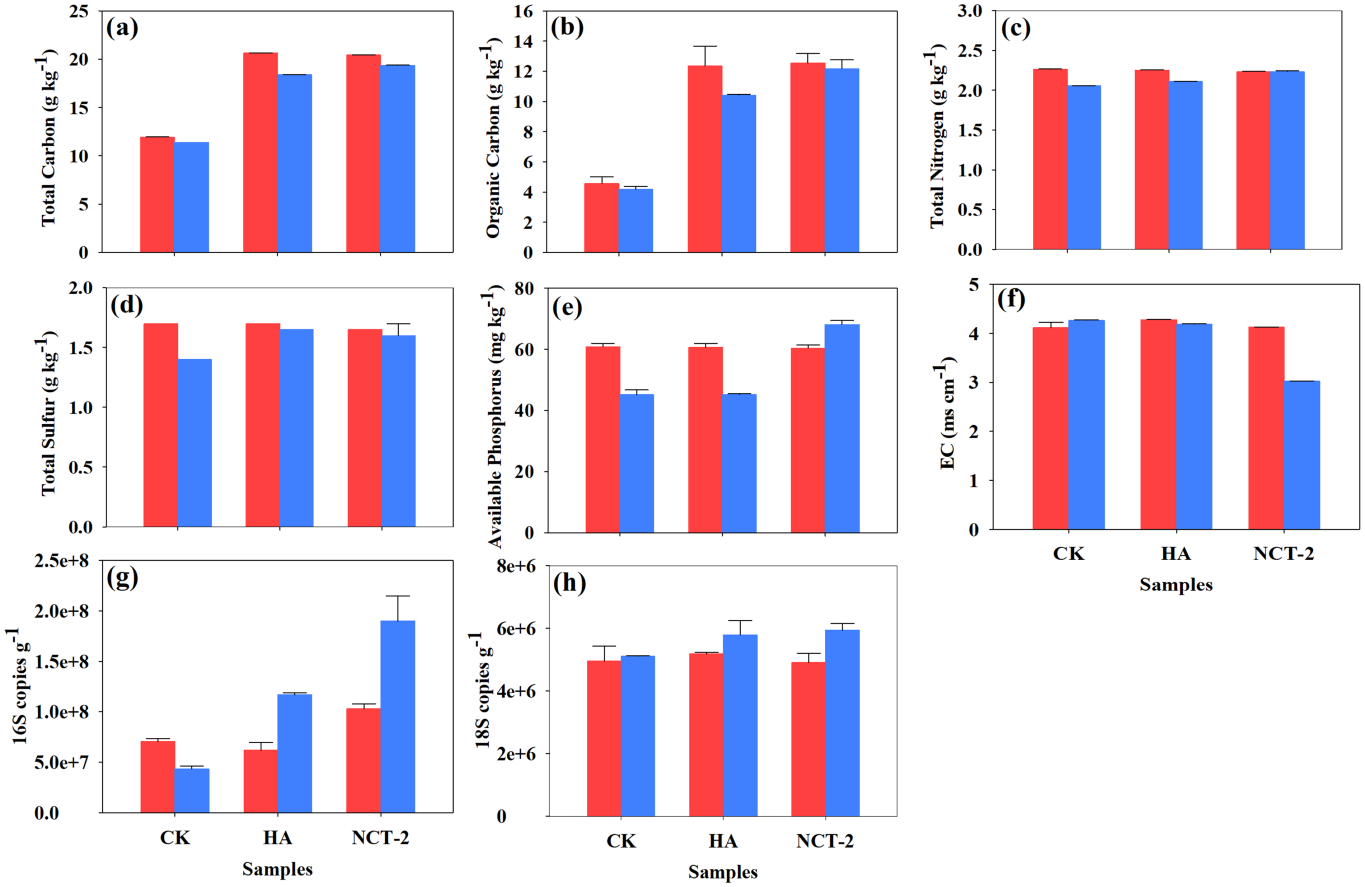
Nutrient indices for test 0 d and 30 d in all soil samples. **(a)** Total carbon content; **(b)** Organic carbon content; **(c)** Total nitrogen content; **(d)** Total sulfur content; **(e)** Available phosphorus content; **(f)** Electrical conductivity; **(g)** 16S number of copies; **(h)** 18S number of copies. Red and blue are the nutrient content on test day 0 d and 30 d, respectively.

In this study, soil nutrients were measured to evaluate the improvement effect and application prospects of microbial agents. At 0 d of the experiment, the total soil carbon of the humic acid (HA) and NCT-2 agent treatments (NCT-2) was significantly higher than that of the control group (CK) (*p* < 0.05) (Figure 4a). After 30 d of the experiment, soil total carbon treated with NCT-2 agent was lower than 0 d and higher than that of humic acid treatment (Figure 4a). This indicated that humic acid increased the total carbon at 0 d. The change of organic carbon is similar to that of total carbon (Figure 4b). The higher TC and TOC in HA and NCT-2 treatments at 0 days reflect carbon inputs from the amendments themselves, whereas subsequent changes (30 d − 0 d) quantify treatment-induced nutrient dynamics. At 30 d, the total nitrogen of NCT-2 agent treatment was consistent with 0 d and higher than that of humic acid treatment and control group (*p* < 0.05) (Figure 4c). Compared with 0 d, there was no significant change in total sulfur after 30 d of treatment with humic acid and NCT-2 agent, but both were higher than the control group (*p* < 0.05) (Figure 4d). Meanwhile, NCT-2 agent significantly increased soil available phosphorus (*p* < 0.05) (Figure 4e). These indicated that NCT-2 agents may reduce soil nitrogen loss, decrease sulfur loss, and increase phosphorus dissolution. In addition, NCT-2 agent significantly reduced soil conductivity (*p* < 0.05) (Figure 4f).

This study found that NCT-2 agent significantly increased the 16S copy number, indicating that this agent significantly increased the number of bacteria in the soil (*p* < 0.05) (Figure 4g). The 18S copy number in the soil was significantly increased by humic acid and NCT-2 agent (*p* < 0.05), and the growth was more significant in the NCT-2 agent treatment. These results indicated that NCT-2 agent can increase the number of soil bacteria and fungi in the process of improving secondary saline soil (Figure 4h).

### 3.4 NCT-2 agent changes vegetable quality

The NCT-2 agent significantly increased the fresh weight of lettuce underground part and overground part (*p* < 0.05). Compared with control group, the fresh weight of the overground part increased by 51.46%, and the fresh weight of the underground part increased by 58.99% (Table 3). Compared with humic acid treatment, the fresh weight of overground part increased by 35.89%, and the fresh weight of underground part increased by 34.44% (Table 3). Therefore, it indicated that NCT-2 agent can increase lettuce yield.

**Table 3:** Effect of NCT-2 agent on vegetable quality.

Treatments	CK1	HU2	NCT3
Fresh weight of overground part (FW) (g)	34.92 ± 2.13 c	38.92 ± 1.42 b	52.89 ± 2.42 a
Fresh weight of underground part (UW) (g)	3.56 ± 0.40 c	4.21 ± 0.39 b	5.66 ± 0.20 a
Glucose (g kg^−1^)	14.03 ± 0.15 a	14.03 ± 0.21 a	14.20 ± 0.10 a
Fructose (g kg^−1^)	12.47 ± 0.15 a	12.33 ± 0.21 a	12.33 ± 0.31 a
Sucrose (g kg^−1^)	2.43 ± 0.15 a	2.57 ± 0.06 a	3.03 ± 0.57 a
Protein (mg kg^−1^)	9.30 ± 0.14 a	8.53 ± 0.14 b	6.28 ± 0.17 c
Nitrate (mg kg^−1^)	689.20 ± 1.48 a	676.70 ± 4.76 a	421.90 ± 4.71 b
Total phenol (TP) (mg kg^−1^)	0.66 ± 0.02 a	0.60 ± 0.02 a	0.60 ± 0.03 a
Total flavonoids (TF) (mg g^−1^)	1.18 ± 0.02 b	0.83 ± 0.12 c	1.64 ± 0.03 a
Antioxidant enzyme activity (AEA) (U g^−1^)	0.32 ± 0.003 b	0.14 ± 0.03 c	0.69 ± 0.02 a
Ascorbic acid (AA) (mg kg^−1^)	215.77 ± 9.46 b	228.20 ± 15.17 b	323.13 ± 21.95 a
Chlorophyll A (μg g^−1^)	456.00 ± 5.68 b	476.99 ± 3.38 b	622.83 ± 2.25 a
Chlorophyll B (μg g^−1^)	83.13 ± 2.71 c	102.20 ± 2.09 b	150.70 ± 2.62 a
Carotenoids (μg g^−1^)	125.11 ± 1.68 b	120.02 ± 1.60 b	132.83 ± 2.10 a
Total chlorophyll (TC) (μg g^−1^)	664.25 ± 10.07 b	U699.21 ± 6.45 b	906.36 ± 5.53 a
Cu (mg kg^−1^)	0.87 ± 0.01 b	0.89 ± 0.02 b	1.51 ± 0.13 a
Mn (mg kg^−1^)	8.18 ± 0.37 a	8.405 ± 0.39 a	7.80 ± 0.18 a
Zn (mg kg^−1^)	6.213 ± 0.20 b	6.615 ± 0.17 b	9.39 ± 0.30 a
Fe (mg kg^−1^)	125.75 ± 14.56 b	134.73 ± 3.08 b	160.57 ± 1.61 a
Mg (mg kg^−1^)	757.60 ± 5.80 a	764.57 ± 24.94 a	579.90 ± 14.27 b
P (mg kg^−1^)	246.10 ± 9.20 b	268.37 ± 6.56 b	326.17 ± 5.54 a
K (mg kg^−1^)	3189.67 ± 146.5 b	3413.00 ± 104.84 b	7812.33 ± 73.89 a
Ca (mg kg^−1^)	2517.33 ± 87.39 a	2246.67 ± 67.45 b	1893.00 ± 83.47 c
Na (mg kg^−1^)	2263.00 ± 74.81 a	2242.33 ± 78.14 a	1815.40 ± 21.51 b

There was no significant change in the content of glucose, fructose, and sucrose in vegetables under different treatments (Table 3). NCT-2 agent significantly reduced nitrate and protein in vegetables (*p* < 0.05) (Table 3). This agent had no significant effect on the total phenolic content in vegetables, but significantly increased the total flavonoids, antioxidant enzyme activity, and ascorbic acid (*p* < 0.05) (Table 3). The chlorophyll a, chlorophyll b, carotenoids, and total chlorophyll were significantly increased in NCT-2 agent (*p* < 0.05) (Table 3). In addition, this agent significantly increased the concentrations of Cu, Zn, Fe, P, and K in vegetables, while reduced the concentrations of Mg, Ca, and Na (*p* < 0.05) (Table 3). In summary, NCT-2 agent improved vegetable yield and quality.

### 3.5 Relationship between rhizosphere and root microorganisms and soil nutrients and vegetable quality

**Figure 5:**
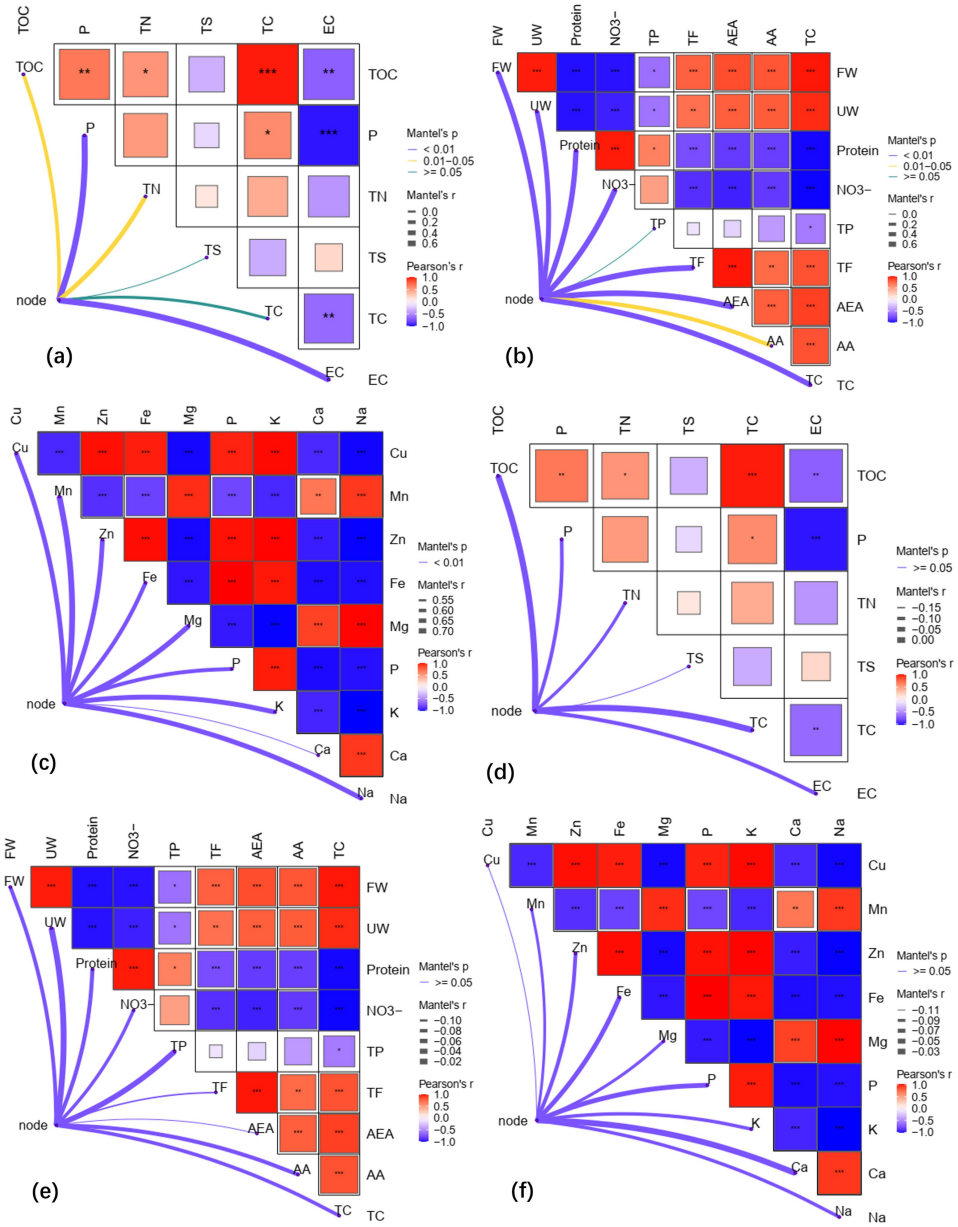
The relationship between microbial community composition and soil nutrient and vegetable quality based on Bray-Curtis distance (Mantel test). **(a)** The relationship between microbial communities and soil nutrients in rhizosphere; **(b)** The relationship between microbial community and lettuce quality in rhizosphere; **(c)** The relationship between microbial community and lettuce mineral nutrients in rhizosphere; **(d)** The relationship between microbial communities of root and soil nutrients; **(e)** The relationship between microbial communities of root and lettuce quality; **(f)** The relationship between microbial communities of root and mineral nutrients. * Means *p* < 0.05, ** means *p* < 0.01, *** means *p* < 0.001. Abbreviations of vegetable quality names are shown in Table 3.

**Figure 6:**
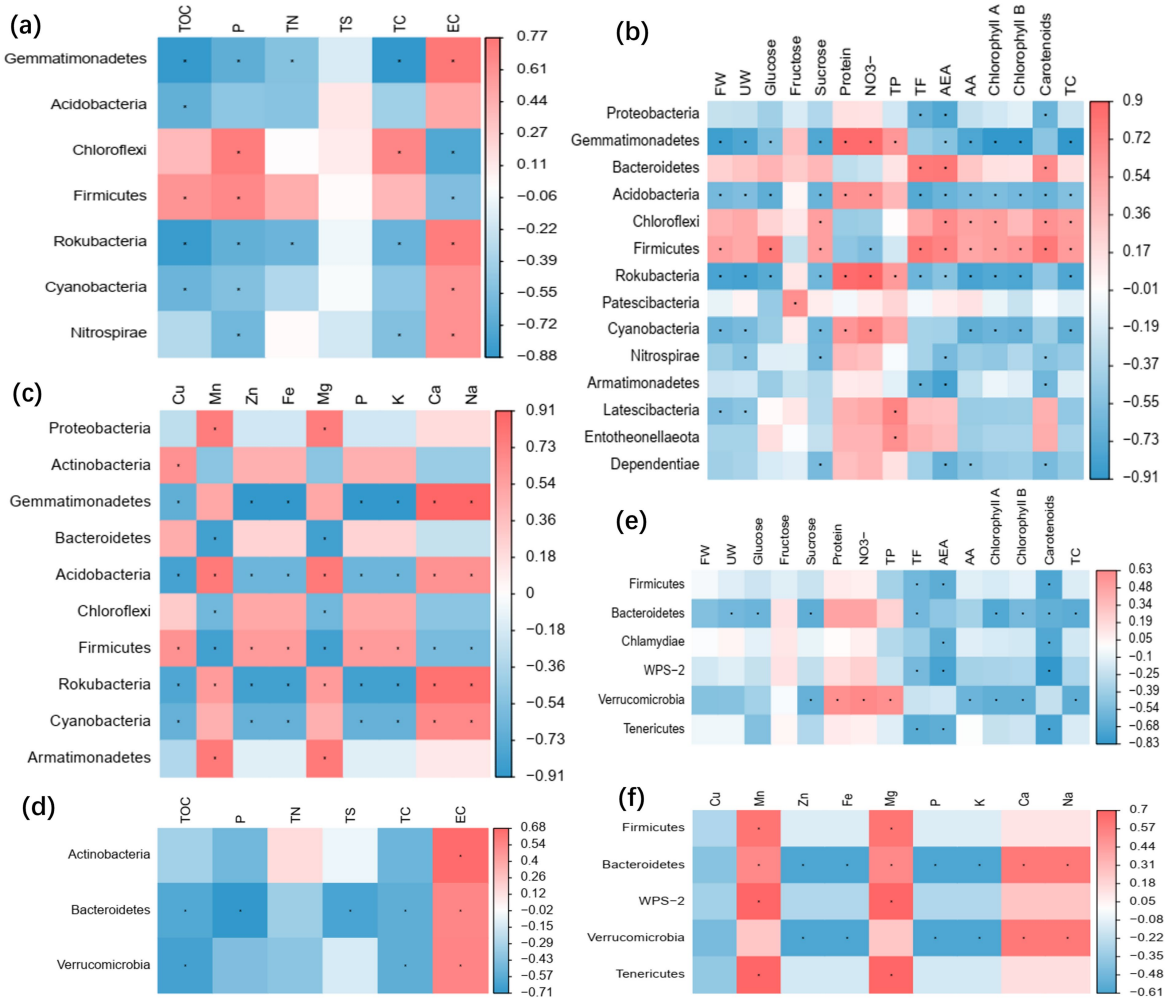
Pearson correlation of microbial communities at the level of phyla with soil nutrients and vegetable quality. In rhizosphere soil, the correlation of bacterial phyla with soil nutrients **(a)**, lettuce quality **(b)**, and mineral nutrients **(c)**, respectively. In root, the correlation of bacterial phyla with soil nutrients **(d)**, lettuce quality **(e)** and mineral nutrients **(f)**, respectively. * means *p* < 0.05, ** means *p* < 0.01, *** means *p* < 0.001. Abbreviations of vegetable quality names are shown in Table 3.

The changes of soil nutrients and vegetable quality caused by microbial agents were closely related to functional microorganisms. Mantel test showed that TOC, P, TN, and EC in soil were significantly correlated with rhizosphere microbial communities (*p* < 0.05) (Figure 5a). *Gemmatimonadetes*, *Chloroflexi*, *Firmicutes*, *Rokubacteria* and *Cyanobacteria* were significantly correlated with soil nutrients (*p* < 0.05) (Figure 6a). Specially, *Firmicutes* and *Chloroflexi* were positively correlated with TOC, P, TN, and TC, and negatively correlated with EC (Figure 6a). FW, UW, protein, NO_3_^−^, TF, AEA, AA, and TC of lettuce were significantly correlated with rhizosphere microbial communities (*p* < 0.05) (Figure 5b). *Gemmatimonadetes*, *Acidobacteria*, *Chloroflexi*, *Firmicutes*, and *Rokubacteria* in rhizosphere were significantly correlated with vegetable quality (*p* < 0.05) (Figure 6b). In particular, *Chloroflexi*, *Firmicutes*, and *Bacteroidetes* were positively correlated with the quality of vegetables (Figure 6b). Rhizosphere microbial communities were significantly correlated with vegetable mineral elements, including Cu, Mn, Zn, Fe, Mg, P, K, Ca, and Na (*p* < 0.05) (Figure 5c). *Gemmatimonadetes*, *Acidobacteria*, *Firmicutes*, *Rokubacteria*, and *Cyanobacteria* play a major role (Figure 6c).

Overall, there was no significant correlation between root microbial community and soil nutrients, vegetable quality and mineral elements (Figures 5d–f). However, some bacteria have certain correlation with soil nutrients and vegetable quality. For example, the *Bacteroidetes* and *Verrucomicrobia* phyla in roots are significantly correlated with soil nutrients, vegetable quality, and mineral elements (Figures 6d–f). These results indicated that the composition of microbial communities is closely related to soil nutrients and vegetable quality. Meanwhile, microbial agents had greater influence on the rhizosphere microbial community, and the root microbial community was more stable.

## 4 Discussion

The soil barrier caused by secondary salinization in facility cultivation is one of the most challenging environmental problems, posing a serious threat to humans and the environment (Zhang et al., 2021). Microbial remediation has the characteristics of low input, high yield, high benefit and no pollution. Therefore, this method is very suitable for remediating secondary salinized soil (Wang et al., 2022). However, the improvement of secondary salinized soil mainly focuses on the removal efficiency of specific salts. Soil contains various nutrients, moisture, osmotic pressure, and other conditions, which are natural culture media for microorganisms (Wang et al., 2019). Microorganisms participate in the cycling of various elements in soil. Therefore, it is important to explore the exact effects of microbial application on soil nutrients, vegetable quality and microecology to make microbial remediation more widely accepted and predictable.

### 4.1 NCT-2 agent changed the rhizosphere and root microbial communities

Plants reside in diverse microbial communities, from the rhizosphere to the endogenous layer. These microorganisms come into direct contact with host plants and play a crucial role in plant growth, health, and soil improvement (Pernilla Brinkman et al., 2010). Our results demonstrate that NCT-2 inoculation reshaped rhizosphere and root microbiota composition, likely through synergistic or competitive interactions with indigenous soil microbes during secondary salinization remediation (Castro et al., 2010; Zhang et al., 2018). Previous studies have also found that microbial agents can change the composition of soil microbial communities, including *Proteobacteria*, *Acidobacteria*, *Actinobacteria*, *Gemmatimonadetes*, *Chloroflexi*, *Nitrospirae*, and *Bacteroidetes* (Naiman et al., 2009; García de Salamone et al., 2010; García de Salamone et al., 2012).

Rhizosphere bacteria and endophytes interact closely with plants (Hardoim et al., 2015). They can promote the plant growth and plant tolerance (Santoyo et al., 2016). Therefore, we speculated that the enriched microbiota in NCT-2 agent treatment has a specific function. For example, studies have proved that *Proteobacteria* was used to measure the nutrient level of soil, and fertile soil is conducive to the its propagation (Castro et al., 2010; Gottel et al., 2011). *Proteobacteria*, a phylum critical to sulfur and nitrogen cycling, showed differential responses to NCT-2 versus humic acid treatments (Pan et al., 2020). While NCT-2 increased specific *Proteobacterial* genera, it suppressed denitrifying bacteria—consistent with our earlier findings on reduced soil denitrification (You et al., 2021; You et al., 2022a; You et al., 2022b). *Firmicutes*-enriched genera like *Bacillus subtilis* and *Lysobacter*, known for stress resistance, nitrogen fixation, and antimicrobial metabolite production, were positively correlated with NCT-2 application (Zhao et al., 2011; Fu et al., 2018). Therefore, NCT-2 agent can alter the function of soil ecosystems by affecting the composition of microbial communities.

### 4.2 Soil nutrients and vegetable quality were improved by NCT-2 agent

Soil nutrient dynamics directly govern crop productivity, underscoring the need for balanced management (Li et al., 2019). Therefore, changes in soil nutrients and vegetable quality were revealed, which contributed to comprehensively evaluate the effectiveness and application prospects of microbial agents in soil remediation.

NCT-2 agent uses humic acid as the carrier, which is the same as the humic acid contained in humic acid treatment. Therefore, the change of soil total carbon and organic carbon is caused by the carbon source contained in humic acid. Total nitrogen showed that NCT-2 agent could reduce nitrogen loss in secondary salinized soil. Our previous research had demonstrated that NCT-2 agent removed excess nitrate from secondary salinized soils through assimilation pathways. Notably, NCT-2 mitigated nitrogen loss in salinized soil by competing with denitrifiers for substrates, thereby curbing nitrous oxide emissions (You et al., 2021). In this study, NCT-2 agent significantly increased the available phosphorus in soil. This showed that the strain had a phosphorus solubilization effect, which transformed the residual phosphorus in the soil into available dissolved phosphorus (Maji et al., 2017). Soil analysis revealed significant increases in available phosphorus, consistent with NCT-2’s genomic capacity for organic acid/phosphate metabolism (Wang et al., 2020). Moreover, NCT-2 agent can maintain the sulfur content in secondary salinized soil.

Microbial biomass carbon, nitrogen, and phosphorus are vital plant nutrient sources (Gentsch et al., 2020). The amount of soil bacteria and fungi was significantly increased under the treatment of NCT-2 agent. According to ecological network analysis, it was found that NCT-2 strain had synergistic or competitive effects with soil microorganisms (You et al., 2022a). This interaction relationship will increase or decrease the number of some microorganisms. NCT-2’s enrichment of soil bacteria and fungi likely results from net synergistic interactions outweighing competitive exclusion (Coyte et al., 2015).

The economic value of microbial agent can be evaluated by analyzing the effect of the agent on vegetable yield. The increase of fresh weight in overground part and underground part indicated that NCT-2 agent increased the yield and root activity of lettuce. Lettuce yield improvements (fresh weight, root activity) correlated with NCT-2’s root colonization and nutrient uptake facilitation (Chu et al., 2018). NCT-2 agent can promote chlorophyll and carotenoids in vegetables. This suggested that the bactericide may affect the photosynthesis of vegetables. This agent reduced the protein and nitrate content of vegetables. In soil and plants, nitrate can be converted into organic nitrogen and enter amino acid metabolism for the synthesis of amino acids and proteins (Kuypers et al., 2018). The significant reduction of nitrate by NCT-2 agent may be the main reason for the decrease in protein content (You et al., 2022a). The increase of ascorbic acid, total flavonoids and the activities of antioxidant enzymes in vegetables indicated that the agent could promote the quality of vegetables (Muthusamy et al., 2019). The concentrations of Cu, Zn, Fe, P, and K were increased by NCT-2 agent, while the concentrations of Mg, Ca and Na were decreased. Previous studies have shown that mineral concentrations may decrease due to dilution effects caused by increased biomass (Dong et al., 2018). Divergent mineral trends—increased Cu, Zn, Fe, P, K versus decreased Mg, Ca, Na—suggest NCT-2 modulates ion transport beyond simple dilution or transpiration effects.

The effect of microbial agents on soil nutrients and vegetable quality is a complex process, and there are many possible reasons for this phenomenon. The addition of exogenous microorganisms can alter the composition of the original microbial community in soil, and may also recruit or exclude other microorganisms through microbial secretions. This creates new competition or collaborative relationships among microorganisms, which changes the microbial community structure and the ability to metabolize substances (Kiboi et al., 2018). Meanwhile, *Bacillus megaterium* is an important phosphate solubilizing bacterium. The metabolism of substances by microbial agents themselves is also one of the reasons for changes in soil nutrients (Maji et al., 2017). Furthermore, studies have shown that vegetable quality indicators are easily affected by growth conditions and fertilization, especially ascorbic acid (Muthusamy et al., 2019). Genomic sequencing confirmed NCT-2 strain a Trp-dependent IAA biosynthesis pathway (e.g., *ipdC*, *aldH* genes) (Wang et al., 2020). This aligns with the observed 85.19% increase in root IAA concentration in NCT-2-treated *S. nigrum*, correlating with enhanced root biomass and Cd accumulation (Chi et al., 2022). Similar IAA-driven mechanisms are well-documented in *Bacillus megaterium STB1* (Nascimento et al., 2020). Improved mineral uptake (e.g., K^+^, P) in lettuce leaves (Table 3) further supports nutrient mobilization, a hallmark of PGP bacteria. Elevated antioxidant levels (e.g., antioxidant enzyme activity, phenols, flavonoids) in lettuce suggest NCT-2 alleviates salt stress, potentially via osmolyte synthesis or rhizosphere microbiome modulation (Arkhipova et al., 2007; Zhou et al., 2016; El-Esawi et al., 2018). These results indicated that NCT-2 agent can improve soil environment and vegetable quality.

### 4.3 Change in key microorganisms contribute to the remediation efficiency of microbial agent

Because of the metabolic diversity and the community’s ability to share metabolites, microbial communities are better able to survive in variable environments than individual species (Wang et al., 2018). Core microbiota-soil nutrient correlations highlight functional synergies between NCT-2-enriched taxa and indigenous microbes (Saleem et al., 2019). In complex soil environments, the impact of specific populations on ecosystem function is not independent of other species. On the contrary, countless positive and negative interactions between different species drive the functionality of the entire ecosystem (Banerjee et al., 2019). These interactions of soil microbial communities may lead to synergies, symbiosis, mutual benefit, parasitism, or competition (Hansen et al., 2007). The NCT-2 agent altered the composition of microbial communities in the rhizosphere and roots. This means that the microbial agent may increase the number of microorganisms with specific functions. This is also demonstrated by the increase in the number of soil microorganisms in this study. Recent studies have shown that the high complexity of microbial interactions in soil can help improve ecosystem function (Banerjee et al., 2019). This is consistent with our previous study, which found that NCT-2 agent was significantly associated with indigenous microorganisms in secondary salinized soils (You et al., 2022a).

The close relationship between rhizosphere and root microorganisms and plants often leads to highly coevolutionary reciprocal interactions. These microorganisms obtain rich nutrients and safe habitats from soil and plants to grow and protect themselves (Reinhold-Hurek and Hurek, 2011). NCT-2’s recruitment of functionally diverse taxa (e.g., nitrogen fixers, phosphate solubilizers, biosurfactant producers) mirrors coevolutionary plant-microbe partnerships that enhance nutrient cycling and stress tolerance (Santoyo et al., 2016). Rhizosphere and endophytic microorganisms can provide nutrient metabolism or material degradation capabilities, thereby promoting metabolic activity in the rhizosphere and endophytic layer (Weyens et al., 2009). In addition, rhizosphere and endophytic microorganisms can produce indole-3-acetic acid, cytokinin, and gibberellin, including *Serratia*, *Enterobacterium*, *Acinetobacter*, *Agrobacterium*, *Bacillus*, *Clostridium*, and *Klebsiella* (Waqas et al., 2015). Perhaps more importantly, these microorganisms produce biosurfactants and colonize the rhizosphere and roots. They enhance substance metabolism by directly releasing biosurfactants into the rhizosphere (Thijs et al., 2016). Therefore, the change of rhizosphere and root microbial communities by NCT-2 agent is helpful to improve the remediation efficiency of secondary salinized soil.

## 5 Conclusion

The potential and application prospect of NCT-2 agent to remediate secondary salinized soil were evaluated in this study. This study establishes that a single *Bacillus* strain can synchronously address salinization-induced nutrient imbalances, enhance crop quality, and reprogram keystone microbial consortia. First, NCT-2 agent improved soil nutrients by reducing nitrogen loss and EC values and increasing sulfur, available phosphorus, bacteria and fungi. Secondly, NCT-2 agent increased the yield and quality of vegetables, including vegetable fresh weight, total flavonoids, ascorbic acid, the activity of antioxidant enzymes, chlorophyll and carotenoids. The dual agroecological benefit—yield enhancement (higher biomass) and nutritional fortification (ascorbic acid increase)—positions NCT-2 as a multifunctional inoculant surpassing conventional nitrate-focused bioremediators. Finally, the composition of microbial communities in the rhizosphere and root of lettuce was altered by microbial agent, including *Latescibacteria*, *Bacteroidetes*, *Firmicutes*, *Acidobacteria*, and *Acetobacter*. Crucially, rhizosphere and root microbiome restructuring revealed NCT-2-driven enrichment of stress-resilient taxa and functional synergists, which correlated mechanistically with soil nutrient fluxes and plant antioxidant capacity. Therefore, this study revealed the effects of microbial agent on soil nutrients, vegetable quality and yield, and root microecology. The adaptability and remediation efficiency of microbial agents strongly demonstrate the applicability of rhizosphere colonizing agents for soil remediation.

## Data Availability

The raw reads were deposited into the NCBI Sequence Read Archive (SRA) database, accession number: PRJNA1081162.
