# Supplementary material for: Bacillus megaterium NCT-2 agent alters soil nutrients, vegetable quality, and root microecology in secondary salinized soil
Source: Front Microbiol. 2025 Apr 22;16:1543933. doi: 10.3389/fmicb.2025.1543933 (PMC12052794; doi:10.3389/fmicb.2025.1543933)
Supplement: Supplementary file 1 [file Supplementary_file_1.docx]

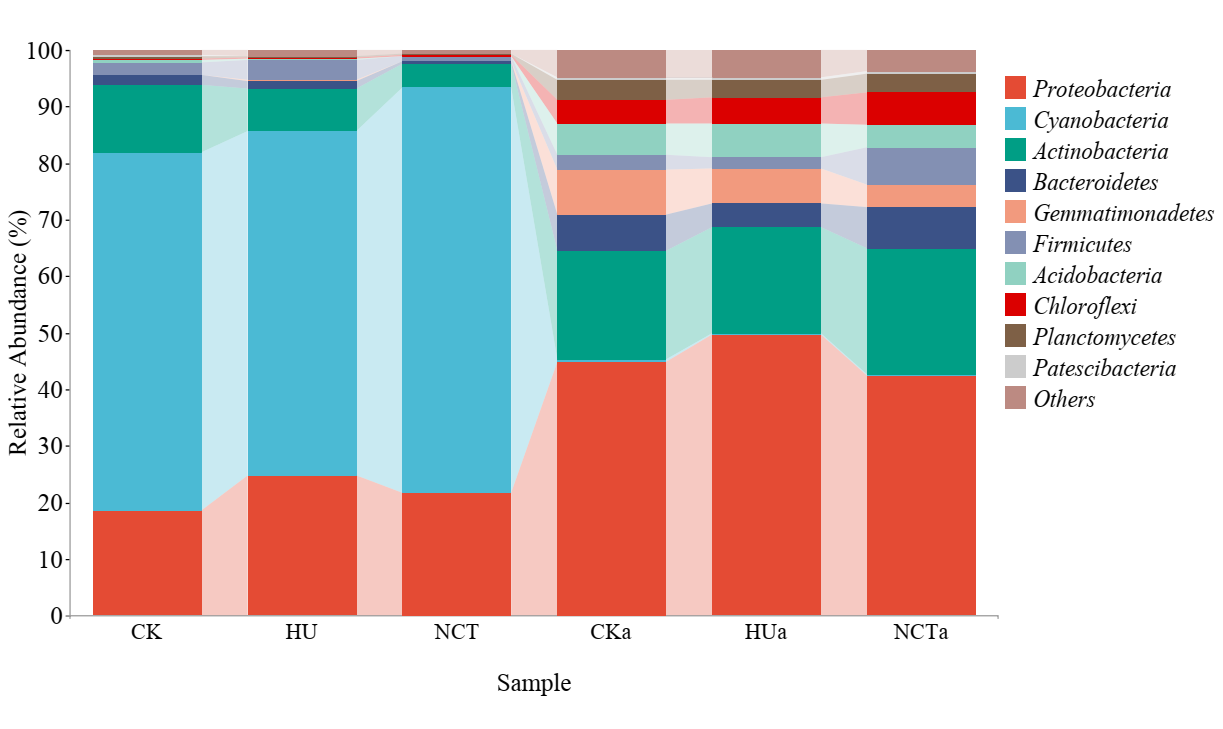


**Supplementary Figure 1** Microbial community composition in rhizosphere soil and root at the level of phyla.
