# Supplementary material for: Bacillus megaterium NCT-2 agent alters soil nutrients, vegetable quality, and root microecology in secondary salinized soil
Source: Front Microbiol. 2025 Apr 22;16:1543933. doi: 10.3389/fmicb.2025.1543933 (PMC12052794; doi:10.3389/fmicb.2025.1543933)
Supplement: Supplementary file 2 [file Table_1.docx]

**Supplementary materials and methods**

**2.1 Soil properties**

Soil moisture was measured by drying the soil subsamples at 105℃ for 48 h. Total C and N contents were analyzed by an elemental analysis coupled to an isotope mass spectrometer using air dried, finely ground soil. Total P content was analyzed by Spectrophotometric determination after alkali fusion. Soil pH was determined on air dried soil at a soil: water ratio of 1:2.5 using a pH meter (Leici Ltd., Shanghai, China). EC values were determined on air dried soil at a soil: water ratio of 1:5 by a conductivity meter (ThermoFisher, Germany). NH_4_^+^ and NO_3_^-^ were extracted with 2 M KCl at a soil/extractant ratio of 1:5 after shaking for 60 min at 250 rpm and 25℃, and analysed on a CleverChem ONE spectrophotometer (Alliance company, France).

**2.2 Quantitative real-time PCR (*q*PCR)**

Each soil sample was freeze-dried for 36 hours using BenchTopPro lyophilizer (Virtis, BenchTopPro, USA) within 24 hours after sample collection. PowerSoil DNA separation kit (Mo-Bio, Carlsbad, CA, USA) was used to extract DNA from 250 mg (dry weight) soil samples, as the manufacturer’s recommended. DNA concentrations were measured using a Nanodrop spectrophotometer ND-1000 (Thermo Fisher, Pittsburg, PA, USA) and purity was measured using polyacrylamide gel electrophoresis. The SYBR Green I fluorescence quantitative PCR kit from TaKaRa and StepOneTM fluorescence quantitative PCR instrument from ABI (Applied Biosystems, USA) were used to conduct the QPCR. The total volume of the QPCR reaction is 20 μL, including 10 μL TB Green Premix Ex Taq II (Takara II, Dalian, China), 0.4 μL ROX Reference Dye, 1 μL 20-fold diluted DNA, 0.8 μL of each primer (10 mM) and 7 μL sterile water.

The16S and 18S genes were identified by *q*PCR in the StepOnePlus Real-time PCR System (Bio-Rad, Hercules, CA, USA). Calibration curves for each gene were obtained from serial dilutions of pMD18-T plasmid templates containing cloned gene fragments of the respective genes. The copies of each standard plasmid were calculated according to the equation described by Nathani et al. (2013).

The equations are as following:

$$Gene copy number (copies/\mu L)=\frac{6.02\times{10}^{23}\times C\times{10}^{-9}\times V}{660\times L}$$

where C is the concentration of recombinant plasmids (ng mL^-1^), V is the total volume of target DNA (mL), L is the full length of recombinant plasmid (bp).

The results of *q*PCR detect an unknown sample can be automatically converted into the copy number of the target gene using the thermocycler instrument. The correlation coefficient (R^2^) of all standard curves was greater than 0.990. The amplification efficiency range is 90~127.0%. The specific amplification was verified by melting curve and agarose gel electrophoresis. The primers and reaction conditions of *q*PCR were shown in Table S1. All samples were run for triplicate with a standard curve and a negative control.

**2.3 Vegetable quality determination**

**2.3.1 Determination of ascorbic acid**

Fresh vegetables were ground into a homogenate. Ascorbic acid was extracted by adding oxalic acid solution. Then, color developing substance and xylene was added, respectively. The absorbance at 500 nm was measured by microplate reader, and the ascorbic acid content of the extract was calculated (Azam et al., 2013).

**2.3.2** **Determination of total phenols and flavonoids**

Total phenols and flavonoids were extracted from 1% hydrochloric acid-methanol mixed solution. The absorbance of the extract was determined at 760 nm, and the total phenol content was calculated (Deng et al., 2013). AlCl_3_ colorimetric method was used to determine the content of flavonoids in vegetables. The 500 µL extract was transferred into a test tube with 1.5 mL methanol, 0.1 mL 10% AlCl_3_, 0.1 mL 1 M potassium acetate, and 2.8 mL distilled water. After standing at room temperature for 30 min, the absorbance of the reaction mixture was measured by microplate reader and the flavonoid content was calculated (Sarker and Oba, 2018).

**2.3.3** **Fructose, glucose, sucrose and total soluble sugar**

The content of total soluble sugar in vegetables was determined by phenol-sulfuric acid colorimetry. Distilled water was used to extract soluble sugar. Then, 0.09 g mL^-1^ phenol and concentrated sulfuric acid were added to the extraction solution. The absorbance of the solution at 485 nm was determined and the soluble sugar content was calculated. Fructose, glucose and sucrose were determined by liquid chromatography. 0.4g lettuce sample was put into 250 mL conical bottle and extracted with 100 mL ultra-pure water in ultrasonic cleaning machine for 10 min. After transfer to a 50 mL centrifuge tube, the extracts were centrifuged at 10000 rpm for 5 min. The supernatant was prepared and then the content of sugar was determined by liquid chromatography (Wang et al., 2021).

**2.3.4** **Protein content and antioxidant enzyme activity**

The protein content and antioxidant enzyme activity were determined by kit. The lettuce was ground in a 0.9% sodium chloride solution at a ratio of 1:9 (weight: volume). The absorbance of the sample was measured by microplate reader according to the instructions.

**2.3.5** **Determination of nitrate content**

Fresh vegetables were washed and ground into a homogenate. The 10g homogenate was put into a 100ml beaker, and 50ml of 70-80 ℃ hot water was added. It was boiled for 15 minutes. After filtration, extract was collected. Nitrate content was determined by automatic discontinuous chemical analyzer (Yu et al., 2018).

**2.3.6** **Determination of total chlorophyll, chlorophyll a, chlorophyll b and carotenoid content**

The content of light and pigment in leaves was determined by the same volume ratio of ethanol and acetone method. Mature leaves in the same position of 0.2 g lettuce samples were weighed. The leaves were cut into evenly sized pieces and placed in test tubes. A mixture of 10 mL ethanol and acetone was added. The tube was soaked in darkness for 12 hours until the leaves turned white. The absorbance of the extract was determined by microplate reader at 470, 645 and 663 nm, and the light and pigment contents were calculated (Nawaz et al., 2018; Sharma et al., 2020).

**2.3.7** **Mineral element content**

Samples of dried lettuce were ground and sifted through 80 mesh. 0.5 g lettuce sample was added to the digestion tank. Then 5 mL of HNO_3_: HCLO_4_ mixed acid with a volume ratio of 4:1 and 1 mL of pure H_2_O_2_ were added. The digestion was carried out with graphite digester. The solution was transferred to a 50 mL volumetric bottle for constant volume. Finally, the content of mineral elements was determined by inductively coupled plasma emission spectrometer (Khaliq et al., 2019; Mizushima et al., 2019).

**2.4 PCR amplification of 16S rRNA gene**

The PCR amplification of 16S rRNA gene was performed as follows: initial denaturation at 95 ℃ for 3 min, followed by 30 cycles of denaturing at 95 ℃ for 30 s, annealing at 55 ℃ for 30 s and extension at 72 ℃ for 45 s, and single extension at 72 ℃ for 10 min, and end at 4 ℃. The PCR mixtures contain 5 × *TransStart* FastPfu buffer 4 μL, 2.5 mM dNTPs 2 μL, forward primer (5 μM) 0.8 μL, reverse primer (5 μM) 0.8 μL, *TransStart* FastPfu DNA Polymerase 0.4 μL, template DNA 10 ng, and finally ddH_2_O up to 20 μL. PCR reactions were performed in triplicate.

**2.5 Processing of sequencing data**

The raw 16S rRNA gene sequencing reads were demultiplexed, quality-filtered by fastp version 0.20.0(Chen et al., 2018) and merged by FLASH version 1.2.7(Magoč and Salzberg, 2011) with the following criteria: (i) the 300 bp reads were truncated at any site receiving an average quality score of <20 over a 50 bp sliding window, and the truncated reads shorter than 50 bp were discarded, reads containing ambiguous characters were also discarded; (ii) only overlapping sequences longer than 10 bp were assembled according to their overlapped sequence. The maximum mismatch ratio of overlap region is 0.2. Reads that could not be assembled were discarded; (iii) Samples were distinguished according to the barcode and primers, and the sequence direction was adjusted, exact barcode matching, 2 nucleotide mismatch in primer matching.

Operational taxonomic units (OTUs) with 97% similarity cutoff(Stackebrandt and Goebel, 1994; Edgar, 2013) were clustered using UPARSE version 7.1(Edgar, 2013), and chimeric sequences were identified and removed. The taxonomy of each OTU representative sequence was analyzed by RDP Classifier version 2.2(Wang, 2007) against the 16S rRNA database (eg. Silva v138) using confidence threshold of 0.7.

**2.6 Data analysis**

One-way analysis of variance with a 5 % probability (p) followed by Tukey’s multiple comparisons test was used to access the significant differences among all the experimental treatments with IBM SPSS Statistics version 26.0 (Chicago, USA). The figure of soil index was finished by GraphPad Prism version 9.0 (Origin Lab, Berkeley, USA). The high-quality sequences were clustered into OTUs (operational taxonomic units) at 97% sequence identity. The microbial taxonomic composition is drawn through R package version 3.2.0. Among all the treatments, bacterial biomarkers were identified by LEfSe (Linear Discriminant Analysi`s Effect Size) analysis in online Galaxy interface (http://huttenhower.sph.harvard.edu/galaxy/). In addition, the correlation between microbial community structure and functional genes and environmental factors has been analyzed by Pearson through IBM SPSS Statistics 26.0. All results were reported as the mean ± standard deviation.

**References**

Azam, A., Khan, I., Mahmood, A., Hameed, A., 2013. Yield, chemical composition and nutritional quality responses of carrot, radish and turnip to elevated atmospheric carbon dioxide. Journal of the Science of Food and Agriculture 93, 3237-3244.

Chen, S., Zhou, Y., Chen, Y., Gu, J., 2018. fastp: an ultra-fast all-in-one FASTQ preprocessor. Bioinformatics 34, 1-11.

Deng, G.-F., Lin, X., Xu, X.-R., Gao, L.-L., Xie, J.-F., Li, H.-B., 2013. Antioxidant capacities and total phenolic contents of 56 vegetables. Journal of Functional Foods 5, 260-266.

Edgar, R.C., 2013. UPARSE: highly accurate OTU sequences from microbial amplicon reads. Nature Methods 10, 996-1007.

Khaliq, M.A., James, B., Chen, Y.H., Ahmed Saqib, H.S., Li, H.H., Jayasuriya, P., Guo, W., 2019. Uptake, translocation, and accumulation of Cd and its interaction with mineral nutrients (Fe, Zn, Ni, Ca, Mg) in upland rice. Chemosphere 215, 916-924.

Magoč, T., Salzberg, S.L., 2011. FLASH: fast length adjustment of short reads to improve genome assemblies. Bioinformatics 27, 2957-2963.

Mizushima, M.Y.B., Ferreira, B.G., França, M.G.C., Almeida, A.-A.F., Cortez, P.A., Silva, J.V.S., Jesus, R.M., Prasad, M.N.V., Mangabeira, P.A.O., 2019. Ultrastructural and metabolic disorders induced by short-term cadmium exposure in Avicennia schaueriana plants and its excretion through leaf salt glands. Plant Biology 21, 844-853.

Nathani, N.M., Patel, A.K., Dhamannapatil, P.S., Kothari, R.K., Singh, K.M., Joshi, C.G., 2013. Comparative evaluation of rumen metagenome community using qPCR and MG-RAST. Amb Express 3, 1-8.

Nawaz, M.A., Jiao, Y., Chen, C., Shireen, F., Zheng, Z., Imtiaz, M., Bie, Z., Huang, Y., 2018. Melatonin pretreatment improves vanadium stress tolerance of watermelon seedlings by reducing vanadium concentration in the leaves and regulating melatonin biosynthesis and antioxidant-related gene expression. Journal of Plant Physiology 220, 115-127.

Sarker, U., Oba, S., 2018. Response of nutrients, minerals, antioxidant leaf pigments, vitamins, polyphenol, flavonoid and antioxidant activity in selected vegetable amaranth under four soil water content. Food Chemistry 252, 72-83.

Sharma, A., Wang, J., Xu, D., Tao, S., Chong, S., Yan, D., Li, Z., Yuan, H., Zheng, B., 2020. Melatonin regulates the functional components of photosynthesis, antioxidant system, gene expression, and metabolic pathways to induce drought resistance in grafted Carya cathayensis plants. Science of The Total Environment 713, 136675.

Stackebrandt, E., Goebel, B.M., 1994. Taxonomic Note: A Place for DNA-DNA Reassociation and 16S rRNA Sequence Analysis in the Present Species Definition in Bacteriology. Int.j.syst.bacteriol 44, 846-849.

Wang, H., Hu, L., Zhou, P., Ouyang, L., Chen, B., Li, Y., Chen, Y., Zhang, Y., Zhou, J., 2021. Simultaneous determination of fructose, glucose and sucrose by solid phase extraction-liquid chromatography-tandem mass spectrometry and its application to source and adulteration analysis of sucrose in tea. Journal of Food Composition and Analysis 96, 103730.

Wang, Q., 2007. Naive Bayesian classifier for rapid assignment of rRNA sequences into the new bacterial taxonomy. Appl.environ.microbiol 73, 1-10.

Yu, T. H., Hsieh, S.P., Su, C.M., Huang, F.J., Hung, C.C., Yiin, L.M., 2018. Analysis of Leafy Vegetable Nitrate Using a Modified Spectrometric Method. International Journal of Analytical Chemistry 2018, 6285867.
