# Supplementary material for: Bacillus megaterium NCT-2 agent alters soil nutrients, vegetable quality, and root microecology in secondary salinized soil
Source: Front Microbiol. 2025 Apr 22;16:1543933. doi: 10.3389/fmicb.2025.1543933 (PMC12052794; doi:10.3389/fmicb.2025.1543933)
Supplement: Supplementary file 3 [file Table_2.docx]

**Supplementary Table 1** Soil properties (averages ± standard deviations)

| N g·kg^-1^ | Organic N g·kg^-1^ | NO_3_^-^ g·kg^-1^ | NH_4_^+^ g·kg^-1^ | EC (S·m^-1^) | Moisture content (%) | pH 1:2.5 (H_2_O) | P g·kg^-1^ |
| --- | --- | --- | --- | --- | --- | --- | --- |
| 8.3±0.058 | 1.13±0.15 | 160.06±1.76 | 1.35±0.15 | 2.29±0.18 | 12.4±0.41 | 7.19±0.08 | 1.4±0.027 |

**Supplementary Table 2** DNA primers used for QPCR

| Gene name | Primer name | Sequence (5’-3’) |
| --- | --- | --- |
| 16S | 16S -F | ACTCCTACGGGAGGCAGCAG |
|  | 16S -R | ATTACCGCGGCTGCTGG |
| 18S | 18S -F | TTAGCATGGAATAATRRAATAGGA |
|  | 18S -R | TCTGGACCTGGTGAGTTTCC |
